# Low-Grade Urachal Cystadenoma With Abundant Calcification Removed Using Robot-Assisted Laparoscopy: A Case Report

**DOI:** 10.7759/cureus.47209

**Published:** 2023-10-17

**Authors:** Andrew P Kochvar, Grant Bednar, Justin M Albani

**Affiliations:** 1 College of Osteopathic Medicine, Kansas City University, Kansas City, USA; 2 Urology, Kansas City Urology Care, Kansas City, USA

**Keywords:** uro-oncology, urology, robotic surgical procedures, abdominal radiology, urachal cystadenoma, urachal cancer

## Abstract

Neoplasms of the urachus are an extremely rare entity consisting of incompletely obliterated tissue of the urachal canal during embryonic development, which sometimes remains into adulthood in the urinary bladder. The treatment of choice for these entities is surgical excision, which maximizes patient survival should the lesion prove to be malignant. In this case, we describe a 57-year-old female who presented with a one-year history of left lower quadrant pain. The patient underwent robot-assisted surgery to remove the mass, bladder dome, and median longitudinal ligament en bloc without evidence of recurrence to date.

## Introduction

Urachal neoplasms are exceedingly rare tumors of urachal remnants within the anterior abdominal wall. Typically, these occur along the length of the median umbilical ligament, which exists as the obliterated fibromuscular canal that bridges the allantois and urinary bladder during embryonic development [1]. Urachal malformations result from focal areas of incomplete obliteration, occasionally evolving into neoplasia if persistent into adulthood [2].

These tend to fall within two morphologic categories: non-cystic and invasive versus predominantly cystic and low-grade [3]. Most urachal cancers arise from the epithelium as glandular tissue or adenocarcinoma within the urachal remnant [4-5]. Urachal adenocarcinomas are most often found in areas corresponding to the inferior urachus, particularly the bladder dome [2,5].

Diagnostic workup typically involves cystoscopy to demonstrate mass and identify gross invasion of the bladder, which should be followed by radiographic imaging (ultrasound (US) plus computed tomography (CT) or magnetic resonance imaging (MRI)) for mass evaluation, staging, and search for metastasis [6-7]. Calcifications are concerning for urachal carcinoma in such lesions [8]. Here, we present a patient who was found to have a mass concerning for urachal adenocarcinoma that instead was found to be urachal cystadenoma with calcified mucin successfully resected using a robotic-assisted laparoscopic approach.

## Case presentation

**Figure 1:**
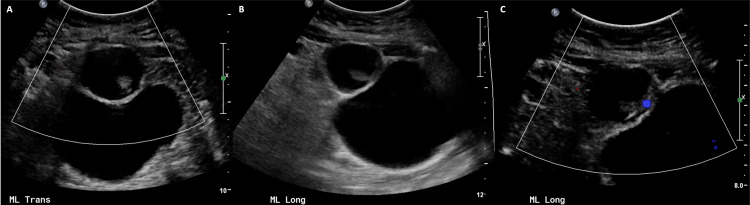
Bladder US in transverse (A) and long (B & C) views demonstrating a well-circumscribed complex cystic lesion attached to the bladder dome. No vascularity is demonstrated within the lesion flow Doppler images (C).

A 57-year-old female with a family history of ovarian, colon, and breast cancer as well as melanoma who was status post open hysterectomy for benign uterine fibroids presented to the clinic for evaluation of a one-year history of left lower quadrant pain. Initial evaluation included a pelvic ultrasound that demonstrated a 3.8 x 3.1 x 3.9 centimeter (cm) cystic lesion in the central pelvis without vascularity on Doppler imaging (Figure 1).

**Figure 2:**
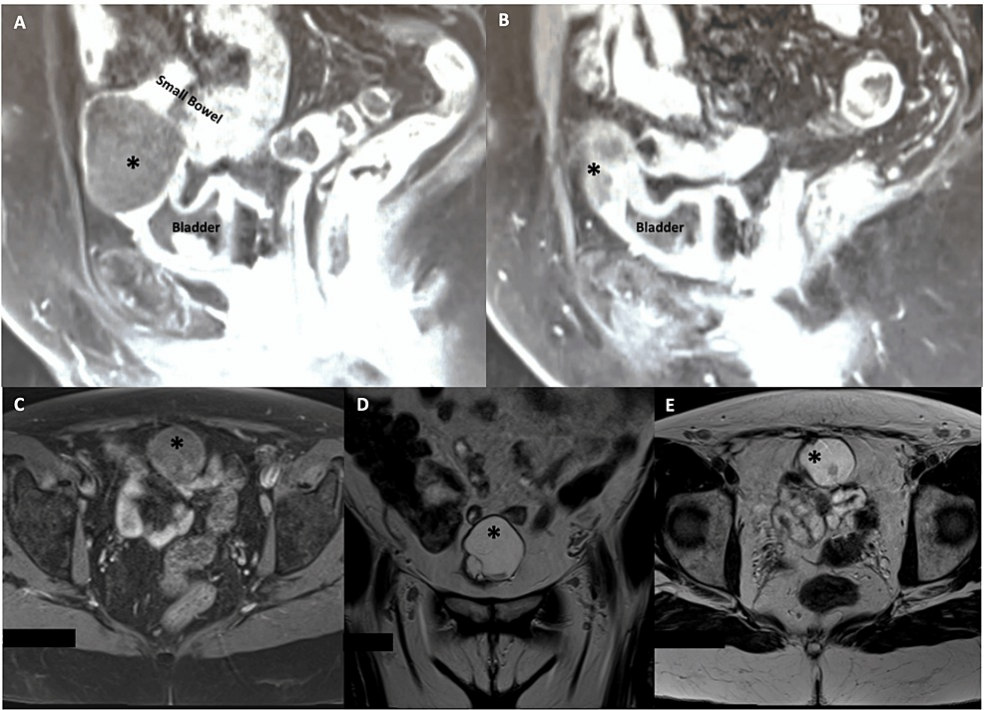
MRI pelvis with and without IV contrast demonstrates the well-circumscribed, multiloculated mass (*) A and B show sagittal sections of T1-weighted fat-saturated post-contrast images of the T1 hypointense lesion (*), demonstrating thickened nodular walls and enhancement in the posteroinferior aspect. T1 fat saturation without contrast axial section (C) demonstrates T1 hyperintensities. D and E show T2-weighted images, which emphasize the mildly heterogeneous, predominantly high T2 signal cystic lesion, focal areas of low T2 signal, and multiple low-signal septations.

Further characterization with MRI pelvis with and without IV gadolinium contrast (Figure 2) noted a 4.3 x 3.6 x 4.2 cm cystic lesion adjacent to the dome of the bladder with thin, low signal septations and nodular wall thickening in the right posteroinferior portion of the lesion measuring 9 x 6 millimeters. No plane between the dome of the bladder was visualized. There was no evidence of a patent urachus, and the patient denied any drainage from the umbilicus.

**Figure 3:**
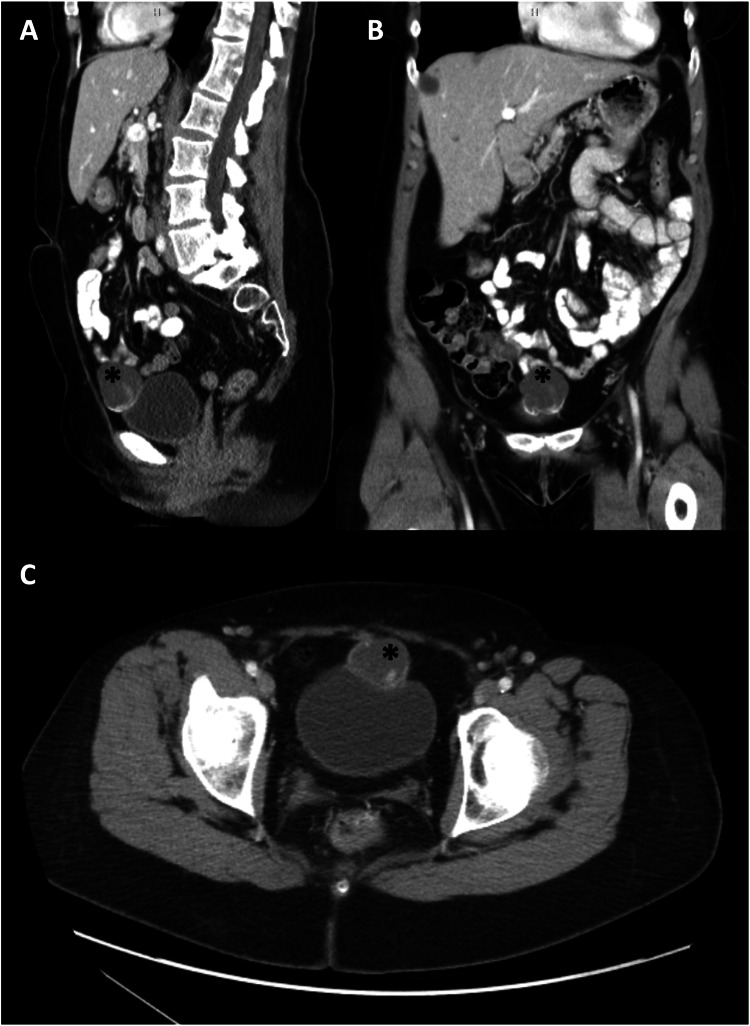
CT chest abdomen and pelvis sagittal (A), coronal (B), and axial (C) slices where the lesion can be visualized The images demonstrate a heterogeneous complex cystic mass lesion (*) with multiple focal hyperdensities and contrast enhancement most prominent at the anterior bladder dome.

She denied any hematuria or voiding complaints. Office cystoscopy demonstrated extrinsic compression from the mass with no visible extension into the bladder. Staging with a CT of the chest, abdomen, and pelvis with contrast noted no evidence of metastasis or other organ involvement (Figure 3).

**Figure 4:**
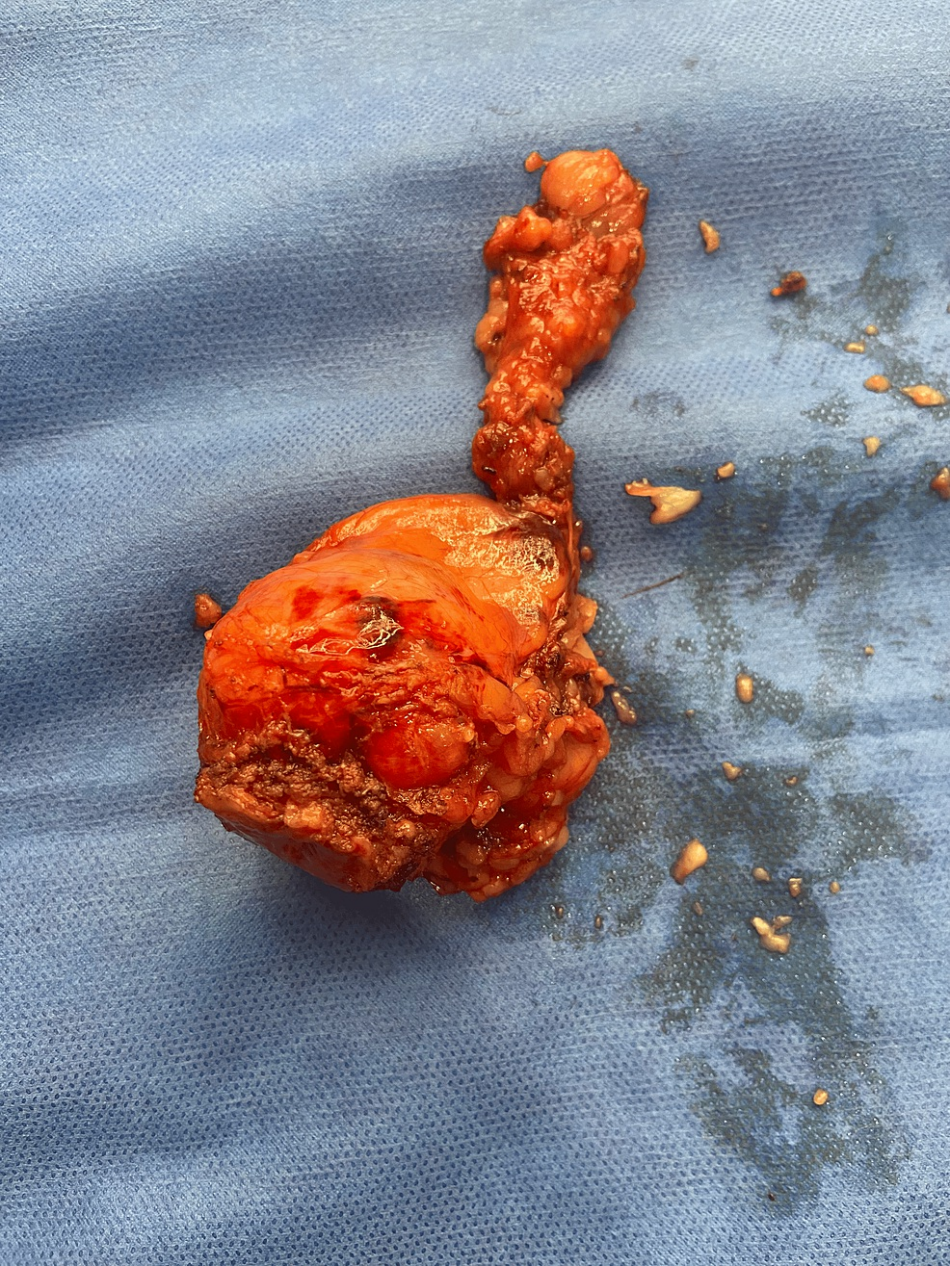
Bladder dome mass, bladder wall, and median umbilical ligament resected en bloc

She underwent laparoscopic robotic-assisted partial cystectomy and excision of the urachal mass, as well as concomitant cystoscopy to facilitate the resection. In order to achieve negative margins, the mass was resected completely and included a portion of the bladder dome with an en-bloc resection of the median umbilical ligaments. The umbilicus was left intact in this case. The resulting mass, which measured 4.7 x 4.4 x 4.2 cm, is shown in Figure 4.

The tissue was sent for processing and pathologic evaluation. The mass grossly demonstrated a multiloculated cyst containing cloudy, mucinous contents. The cyst lining contained focal areas of calcification while the cyst did not demonstrate involvement of the bladder mucosa. Histology confirmed this lesion as a low-grade mucinous cystadenoma of the urachus containing acellular, calcified mucin. No carcinoma was identified in any section.

The patient convalesced without complications and has been without radiographic or cystoscopic evidence of recurrence to date.

## Discussion

Urachal malignancies constitute fewer than 1% of all neoplastic lesions of the bladder, and benign lesions represent an even smaller subset [9-10]. Urachal neoplasms often arise from the pre-existing urachal remnant at the junction between the urachus and bladder [2,4]. A multidisciplinary team is frequently required for proper diagnosis and treatment based on radiologic-pathologic correlates after surgical intervention.

The typical patient with urachal carcinoma presents with hematuria within the sixth decade of life, with a median age between 52 and 59 years, significantly younger than those typically affected by urothelial cancer [11]. Patients will most often present with frank hematuria, which warrants cystoscopy to visualize the anterior bladder and dome to evaluate for malignant bladder tumors, which is the location of 90% of all urachal carcinomas [5,10]. Certain presenting symptoms or imaging features can help inform the differential diagnosis and distinguish between carcinoma versus genitourinary infections. Infection is commonly associated with abdominal pain, bladder wall thickening, and female sex, whereas urachal carcinomas are often associated with hematuria and calcifications on imaging [10]. Our patient presented with a benign lesion containing calcifications and a chief complaint of abdominal pain. Cystoscopy alone may not demonstrate specific bladder abnormality, as seen in our case. This further emphasizes the importance of radiologic imaging and histopathologic correlation for a definitive diagnosis and proper treatment because benign masses often mimic malignant ones.

The low incidence of urachal neoplasms has made definitive consensus guidelines difficult to create, though most evaluations in the literature use similar sequential approaches. US is often used first to characterize the lesion [10,12]. This modality has a high degree of accuracy for diagnosing urachal anomalies [13]. CT and MRI, particularly images in the sagittal plane, are most appropriate for lesions that cannot be fully characterized by US or those that have features concerning for malignancy [13,14]. On MRI, the most concerning features to be aware of include heterogenous T2-hyperintensities within a midline soft-tissue mass that enhances with intravenous contrast [15]. These are also excellent modalities for disease staging.

Solid urachal masses, particularly those with macroscopic calcification, are more likely to be malignant than tumors without calcification although CT has demonstrated low negative predictive value (43%) and specificity (21%) for urachal carcinoma [4,16,17]. Although approximately 50% of the urachal carcinomas imaged preoperatively by CT are solid, a significant minority contains a cystic component [4]. Urachal soft tissue masses with calcifications are generally considered the pathognomonic presentation of urachal carcinoma, though approximately 20% of urachal cysts are known to contain peripheral calcifications [4,8]. Central or peripheral calcification is present in nearly 75% of urachal cancers on CT [8,13].

Currently, there are no consensus guidelines for the staging of urachal neoplasms. Urachal carcinomas historically were staged using the Sheldon Staging System, though more recently the novel Mayo Clinic Staging System has gained popularity and is being utilized as a staging system [4]. Urachal malignancy may be detected using fluorodeoxyglucose-positron emission tomography (FDG-PET)/CT, though it has been shown to be equivalent for staging to CT with IV contrast and, therefore, has limited utility for this entity [18].

Patients with suspected urachal carcinoma require wide surgical excision of the lesion and the remainder of the median umbilical ligament, partial or radical cystectomy, and sometimes bilateral pelvic lymphadenectomy [11]. Partial cystectomy with en-bloc removal of the median umbilical ligament with or without umbilectomy is essential for improving survival in patients with suspected urachal carcinoma [4,19]. An open surgical approach is most commonly used and considered the gold standard though laparoscopy is considered to be a safe alternative [20]. Notably, robot-assisted resection has shown promise as an equally successful approach with no difference in rates of positive surgical margins compared to the open approach and is associated with reduced perioperative morbidity [20]. Our patient presented a unique opportunity to perform robotic surgery with excellent results.

## Conclusions

This case report demonstrates an extremely rare pathology that has only been described within other case reports. Urachal masses require careful evaluation because the ability to distinguish urachal adenocarcinomas from those of primary bladder origin greatly impacts care, surgical approaches for treatment, and quality of life. Our case emphasizes the importance of proper radiologic and surgical management to mitigate any progression of a potential malignancy.
